# Outcomes in Patients with Spinal Metastases Managed with Surgical Intervention

**DOI:** 10.3390/cancers16020438

**Published:** 2024-01-19

**Authors:** Brendan Knapp, Ashwin Govindan, Shalin S. Patel, Kymberlie Pepin, Ningying Wu, Siddhartha Devarakonda, Jacob M. Buchowski

**Affiliations:** 1 Department of Medicine, Division of Oncology, Washington University School of Medicine, St. Louis, MO 63110, USA; bjknapp@wustl.edu (B.K.);; 2 Department of Orthopaedic Oncology, The University of Texas MD Anderson Cancer Center, Houston, TX 77030, USA; 3 Biostatistics Shared Resource, Division of Public Health Sciences, Department of Surgery, Siteman Cancer Center, Washington University School of Medicine, St. Louis, MO 63110, USA; 4 Department of Orthopedic Surgery, Washington University School of Medicine, St. Louis, MO 63110, USA

**Keywords:** spinal metastases, outcomes, retrospective, spine surgery

## Abstract

**Simple Summary:**

Metastases to the spine are associated with significant pain, decreased quality of life, and worse survival in patients with advanced cancer. Management often includes surgical intervention, but identifying patients who may not benefit from surgery remains a critical knowledge gap. We retrospectively evaluated patients with spine metastases who underwent surgery at our institution to identify characteristics predictive of poor outcome. We found patients with additional metastases outside the spine, age > 65 years at surgery, and poor functional status to be factors associated with death at 180 days; patients with these factors and BMI ≤ 30 mg/kg^2^ had worse overall survival. Our findings support multidisciplinary discussions regarding the benefits and risks associated with spinal surgery in patients with these risk factors.

**Abstract:**

Background: Spinal metastases are a significant cause of morbidity in patients with advanced cancer, and management often requires surgical intervention. Although prior studies have identified factors that influence outcomes with surgery, the ability of these factors to predict outcomes remains unclear in the era of contemporary therapies, and there is a need to better identify patients who are likely to benefit from surgery. Methods: We performed a single-center, retrospective analysis to evaluate risk factors for poor outcomes in patients with spinal metastases treated with surgery. The primary outcome was mortality at 180 days. Results: A total of 128 patients were identified. Age ≥ 65 years at surgery (*p* = 0.0316), presence of extraspinal metastases (*p* = 0.0110), and ECOG performance scores >1 (*p* = 0.0397) were associated with mortality at 180 days on multivariate analysis. These factors and BMI ≤ 30 mg/kg^2^ (*p* = 0.0008) were also associated with worse overall survival. Conclusions: Age > 65, extraspinal metastases, and performance status scores >1 are factors associated with mortality at 180 days in patients with spinal metastases treated with surgery. Patients with these factors and BMI ≤ 30 mg/kg^2^ had worse overall survival. Our results support multidisciplinary discussions regarding the benefits and risks associated with surgery in patients with these risk factors.

## 1. Introduction

Spinal metastases are a significant cause of morbidity and mortality among cancer patients and are becoming increasingly common as life-prolonging therapies for metastatic cancer improve [1,2,3]. The optimal treatment strategy for spinal metastases is not well defined and greatly depends on each individual’s clinical situation. Broadly, patients can be treated with radiation therapy (RT), systemic therapy, interventional techniques such as kyphoplasty, or surgery, including en bloc metastases resection, debulking, or stabilization procedures [4,5,6,7,8]. Surgery is typically indicated in patients with spinal instability, neural compression, and pain unresolved by prior interventions [9,10,11,12]. Prior studies have found factors predictive of worse survival after surgery to include functional status, tumor type [4,13,14,15,16,17,18,19,20,21,22,23], the presence of visceral metastases [17,24], comorbidities [15], and perioperative complications [25]. Predicting early mortality following surgery has been less well studied, but sarcopenia, frailty, age, albumin level, and tumor type have been identified as possible predictors of early mortality following surgery [22,26,27,28,29]. However, many of these studies evaluated patients from the 1990s and 2000s, and the utility of these predictors of survival, particularly with the availability of stereotactic RT and other contemporary systemic therapy options—such as immunotherapy—is unclear. Thus, the evaluation of mortality and functional status in patients with spinal metastases treated with modern therapy and surgical techniques is needed, and identifying patients unlikely to benefit from invasive surgery remains a critical knowledge gap.

We aimed to identify patients who are at high risk of poor outcomes following surgical intervention. We hypothesized that patients with risk factors such as (i) poor functional status; (ii) extraspinal metastases; (iii) older age at presentation with spinal metastatic disease (>65 years); and (iv) those who, with increased spinal metastatic burden, are unlikely to derive meaningful survival benefit (defined as >180 days post-surgery and the ability to receive additional post-operative systemic therapy) from surgical intervention [15,17,24,25]. We performed a single-center, retrospective analysis to evaluate this hypothesis, and we report the results here. We found age, extraspinal metastases, and performance status were factors associated with early mortality.

## 2. Materials and Methods

Patients evaluated included those who underwent surgical intervention for spinal metastases at Barnes Jewish Hospital from 1 September 2006 to 1 December 2020. Patients with benign tumors or non-cancerous lesions as well as those with primary bone tumors were excluded. Patients without data available in ClinicalDesktop version (v) 2 (Saint Louis, MO, USA), Allscripts v19.4 (Chicago, IL, USA), or Epic v2021 (Verona, WI, USA) and those < 18 years of age at the time of surgery were also excluded. Charts were reviewed by three research members and audited for accuracy. Information extracted included the age/date of initial diagnosis and surgery, receipt of pre- and post-operative chemotherapy, immunotherapy, and radiation therapy, BMI and ECOG performance status at surgery, tobacco use, the presence of extraspinal metastases, the number of vertebrae involved, and post-operative complications, among other factors. For survival analysis, the tumor types were consolidated into 6 groups: Breast, Lung, Renal, Sarcoma, Hematologic (Multiple Myeloma and Lymphoma), Other Solid (Bladder, Gastrointestinal, Gynecologic, Prostate, Thyroid, Melanoma) and Other (two with head and neck cancer; two with squamous cell carcinoma of unknown primary; and one each with neuroblastoma, neuroendocrine tumor of unknown primary, and thymoma). By consolidating tumor types into six broader categories, we aimed to achieve a more practical sample size, thereby increasing statistical power and enhancing the precision and reliability of our statistical inferences.

The primary outcome was mortality at 180 days. This was a categorical yes/no variable defined as patient status (alive or dead) at 180 days from the date of surgery for spinal metastases at Barnes Jewish Hospital to date of death from any cause, or to the last medical oncology, surgical oncology, or radiation oncology visit if alive. Secondary outcomes included overall survival (OS) (defined as the number of days from initial surgery for spinal metastases at Barnes Jewish Hospital to date of death of any cause) and receipt of additional post-operative therapy, including chemotherapy, immunotherapy, or other targeted therapy.

Data were extracted from Epic v2021, ClinicalDesktop v2, and AllScripts v19.4 and transferred to Excel v2021 (Redmond, WA, USA). Data were then uploaded to REDCap v13 (Research Electronic Data Capture; Nashville, TN, USA) for further analysis. All statistical analyses were performed using SAS version 9.4 (SAS Institute, Cary, NC, USA). Demographic and clinical characteristics were summarized as counts and percentages. Kaplan–Meier (KM) curves were generated to provide unadjusted OS estimates.

Various risk factors for primary and secondary outcomes, including age at diagnosis, age at surgery, tumor type, BMI, the number of vertebrae involved, the presence of extraspinal metastases, ECOG performance status, and pre-operative therapy, were assessed via univariate and multivariate regression models. Stepwise selection was used in the multivariate analyses, where a significance level of 0.3 was required to allow a risk factor into the model, and a significance level of 0.15 was required for a risk factor to stay in the model.

For binary outcomes (i.e., mortality at 180 days and receipt of additional post-operative therapy), logistic regressions were performed. Firth’s penalized likelihood estimation was used to mitigate the bias caused by rare events in the data set. For OS, Cox proportional hazard regressions were performed. Proportional hazard assumption was examined via log of negative log plots and significance tests of time-dependent covariates. No significant violation of the time independence assumption was found.

## 3. Results

A total of 176 patients were identified for our analysis. Among these, 48 patients were excluded from further analysis, including 2 patients who were <18 years of age at the time of surgery, 19 patients with incomplete data in our electronic medical records, 18 patients with benign tumors, and 9 with unknown mortality status. Characteristics of the 128 patients included in the analysis are shown in Table 1 and Supplementary Tables S1–S3. Most patients were white (89%), <65 years at surgery (73%), with BMIs between 20 and 30 mg/kg^2^ (56%) and had ECOG performance status scores of 0 or 1 (73%). Lung cancer was the most common tumor type (24%), followed by renal cell (16%) and breast (12%). Thoracic vertebrae represented the most common location of spinal metastases (71%), and a majority of patients had extraspinal metastases (52%). Most patients had more than one vertebral metastasis (71%). Less than half (44%) of the patients had received cytotoxic therapy prior to surgery, and 46% of patients received preoperative radiation therapy. A combined anterior and posterior approach was the most common surgical technique (57%).

Post-operative infections occurred in 13% of patients, and 27% of patients experienced other post-operative complications. Of the 34 patients with post-operative complications, the most common complications were venous thromboembolism (8/34, 24%), wound-related (24%), worsened neurologic status (24%), and cardio-respiratory related complications (18%). In total, 4 of 128 patients (3%) died within 30 days of the index surgery, and an additional 2 patients died within 30 days of a subsequent surgery. Additional spinal surgeries were performed in 18% of patients, and post-operative radiation therapy was received by 61% of patients. Of 75 patients with detailed neurologic status available, most (95%) were symptomatic prior to surgery, and a majority of patients had stability or improvement in neurologic status acutely post-operatively, as well as at 1–3 months post-operatively (Supplementary Table S4).

In regard to the primary outcome, 30 of 128 patients (23%) died before 180 days. Age > 65 at surgery (*p* = 0.0316), the presence of extraspinal metastases (*p* = 0.0110), and ECOG performance status > 1 (*p* = 0.0397) were associated with an increased risk of mortality at 180 days (Table 2). Age at diagnosis, tumor type, BMI, the number of vertebrae involved, and receipt of pre-operative therapy were not associated with mortality at 180 days. In total, 72 of 128 (56%) patients were able to receive post-operative therapy. Of the evaluated factors, no characteristics were associated with receipt of post-operative therapy, except for tumor type (*p* = 0.0491), with patients with breast cancer having the increased likelihood of being able to receive post-operative therapy (Supplementary Table S5).

**Figure 1:**
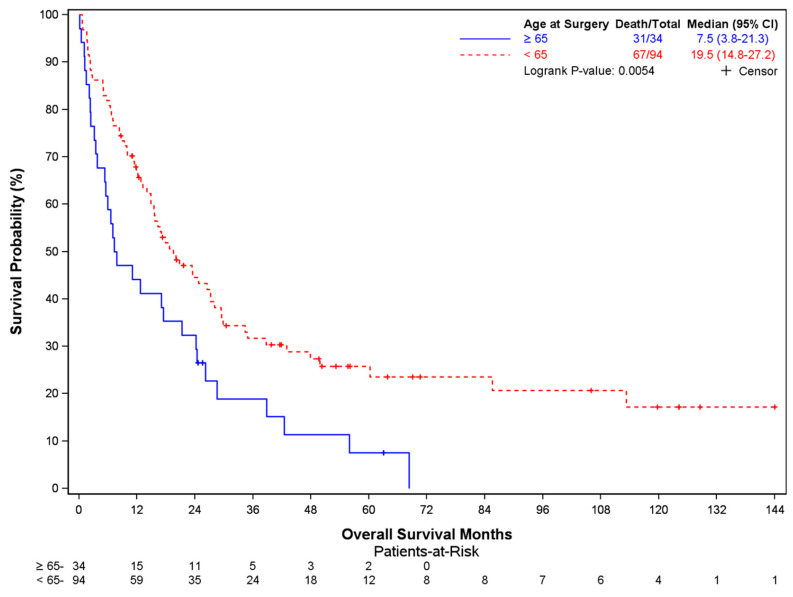
Overall survival in patients stratified by age at surgery.

**Figure 2:**
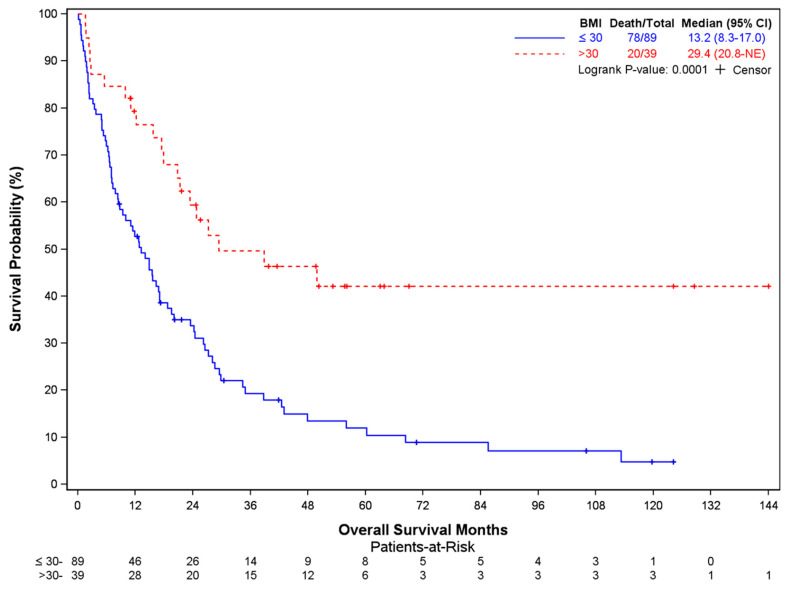
Overall survival in patients stratified by BMI at surgery.

**Figure 3:**
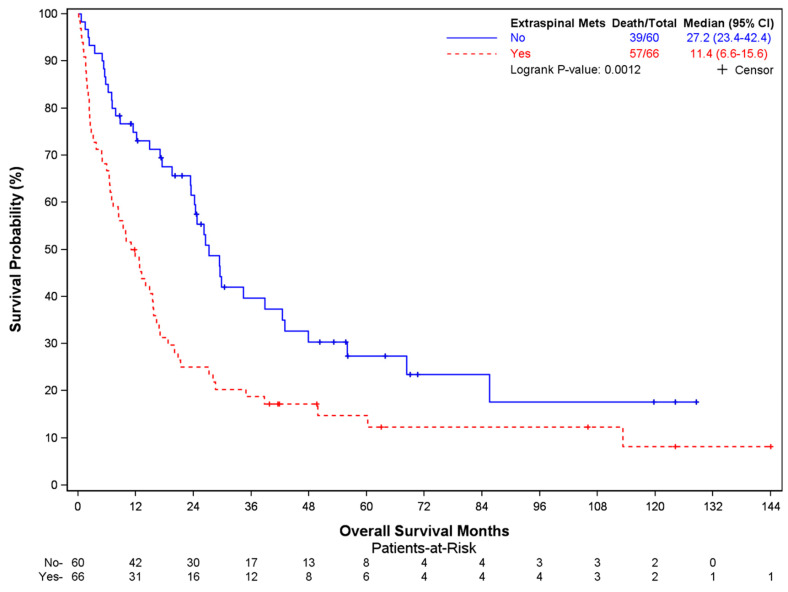
Overall survival in patients stratified by presence of extraspinal metastases.

**Figure 4:**
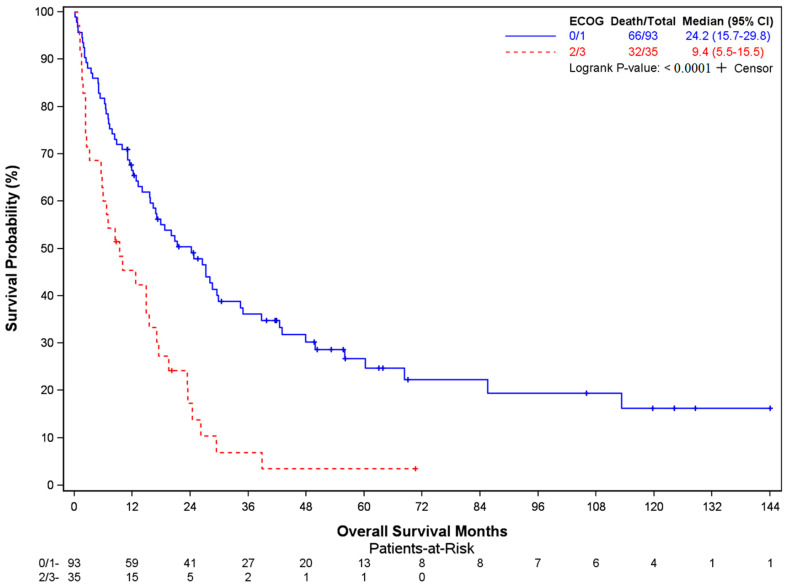
Overall survival in patients stratified by ECOG performance status score.

In total, 98 of 128 patients had died at the time of data cutoff (median OS 17 months, 95% CI: 12.7–23.5 mo). Older age at surgery (*p* = 0.0016), lower BMI (*p* = 0.0008), the presence of extraspinal metastases (*p* = 0.0001), and ECOG performance status >1 (*p* = 0.0006) were factors associated with worse overall survival (Table 3). The median OS was 19.5 months (95% CI 14.8–27.2) in patients < 65 years of age at surgery compared to 7.5 months in patients aged ≥ 65 years (95% CI 3.8–21.3) (Figure 1). Patients with a BMI ≤ 30 mg/kg^2^ showed a median OS of 13.2 mo (95% CI 8.3–17.0), compared to 29.4 mo (95% CI 20.8–NE) in those with BMI > 30 (Figure 2). Patients with extraspinal metastases at the time of surgery displayed worse survival, with a median OS of 11.4 mo (95% CI 6.6–15.6) versus 27.2 mo (95% CI 23.4–42.4) (Figure 3). Similarly, patients with an ECOG performance status >1 had a much worse prognosis (median OS 9.4 mo, 95% CI 5.5–15.5) than those with an ECOG performance status of 0 or 1 (median OS 24.2 mo, 95% CI 15.7–29.8) (Figure 4). Tumor type was not identified as a significant risk factor in the multivariate Cox model, although unadjusted Kaplan–Meier log-rank test results showed patients with hematologic malignancies having a better prognosis compared to patients with other cancers (median OS NR, 95% CI 26.1–NE), particularly lung (median OS 9.4 mo, 95% CI 6.4–15.6) (*p* = 0.0030) (Supplementary Figure S1). Age at diagnosis, the number of vertebrae involved, and receipt of pre-operative therapy were not associated with survival (Supplementary Figures S2–S4).

## 4. Discussion

Widely used scoring systems for spinal metastases to predict survival include the Tomita, modified Bauer, and Tokuhashi scores. The Tomita scoring system uses the rate of growth of the malignancy, presence of visceral metastases, and the presence of solitary vs. multiple bone metastases [30]. The Tokuhashi score incorporates performance status, the number of vertebral metastases, the number of extraspinal metastases, visceral metastases, primary tumor type, and neurologic status [31]. The modified Bauer score includes the presence of visceral metastases, tumor type, and number of vertebral metastases [32]. However, both the Tomita and Tokuhashi scores showed low accuracy in predicting 6-month survival in a meta-analysis by Lee et al. [33], and the utility of these scores in the modern area is unclear, especially as they were developed using patients from the 1980s–2000s, prior to contemporary therapeutics. A more recently developed tool is the New England Spinal Metastasis Score (NESMS) [24]. It uses the modified Bauer score, serum albumin (cut off of 3.5), and ambulatory status (impaired versus not), and was shown to be predictive of 1-year and 30-day mortality [34]. While the score was derived from patients treated from 2007 to 2013, it has since been prospectively validated as a predictor of 6-month and 1-year mortality in a study of 180 patients treated between 2017 and 2018 [35]. The NESMS was shown to be able to differentiate survival to a higher degree than the Tokuhashi, Tomita, and Spinal Instability Neoplastic Scores in a prospective study of 202 patients [36] but did not meet a pre-defined clinical utility threshold in a separate retrospective study by Garza-Ramos et al. [37], and the optimal predictive tool remains to be defined.

We sought to identify patient- and disease-related risk factors that could predict poor early outcomes despite surgical intervention in patients with spinal metastases. We found that age at surgery, the presence of extraspinal metastases, and ECOG performance status at the time of surgery were associated with an increased likelihood of mortality at 180 days. In regard to overall survival, older age at surgery, lower BMI, extraspinal metastases, and ECOG performance status were associated with worse overall prognosis on multivariate analysis. Tumor type was associated with worse prognosis on univariate analysis, but not multivariate. The presence of extraspinal/visceral metastases and older age at surgery (>65 years) was associated with worse 180-day and overall survival, consistent with other retrospective studies [4,13,14,15,16,17,19,20,38,39]. Receipt of pre-operative therapy has had varying prognostic value in prior studies and was not predictive of survival in our cohort [13,18,19,38], nor was the number of vertebral bodies involved [16,18,19,20,21,38]. Generally, patients with worse preoperative functional status have worse overall survival [16,18,19,25,40], and this was also noted in our study. Patients with lung cancer appeared to have a particularly poor prognosis in our cohort, consistent with other studies, which may be secondary to the relative lack of treatment options for advanced lung cancer compared to other malignancies [4,14,15,16,17,18,19,20,21,22,23,41]. It is well known that patients with cachexia or significant weight loss have poor prognosis and patients with a low BMI undergoing surgery, especially those underweight, are at higher risk for worse outcomes [22,42,43,44], and we found that patients with a BMI > 30 had improved survival compared to those with a lower BMI. A recently published systematic review of 61 studies evaluating pre-operative variables on post-operative outcomes included 22,335 patients. The authors found factors predictive of worse survival amongst prior studies to include older age, lower BMI and weight loss, male sex (in 5 studies), smoking status, worse neurologic function, poor performance status, increased systemic disease burden, and low albumin (in five studies), among other factors. Most of these studies were retrospective in nature, like ours [3].

While prior studies have evaluated overall survival in this patient population, fewer have evaluated early mortality following surgery. The NESMS has been shown to predict 30-day mortality and major systemic complications in a retrospective study of 776 patients published in 2016 [34]. Anzuategui et al. found tumor growth rate (adopted from Tomita et al.’s model [30]), age ≥ 70, presence of serious comorbidity, and lymphocyte count < 1000 cells/µL to be independently associated with increased 30- and 90-day mortality [28]. Frailty and sarcopenia have been previously identified as predictors of 90-day mortality by two groups [26,29]. Taken together, functional status, visceral metastases, age, and perhaps tumor type should be considered as predictors for early mortality. Importantly, it is difficult to identify a length of survival following surgery that determines “benefit”. We chose 180 days as it is the cutoff for hospice eligibility by the Centers for Medicare & Medicaid Services. Determining who may recover from surgery to receive additional systemic therapy remains challenging, and no factors were found to be predictive in our cohort.

Our study has several limitations, including those inherent to retrospective cohort studies such as the use of a single-tertiary-center population. Additionally, our study included a heterogeneous group of tumors. Some of the included tumor groups had low numbers of patients, leading to underpowered subgroup analysis with wide confidence intervals, limiting conclusions for a tumor type’s impact on prognosis. Patients with lung cancer were over-represented, at 24% of the patient population. Neurologic status, especially post-operatively, was not available for all patients, and was not formally included in our analysis. The absence of an external validation cohort or comparison to a non-operative cohort is an additional weakness, as was the lack of direct comparison to other scoring systems due to data availability limitations. Finally, this cohort included patients who underwent surgery, thus only including those who were deemed “operative” by the surgical team and does not represent all patients with spinal metastases. Advantages of our study include detailed descriptive patient characteristics, especially pre- and perioperatively. Additionally, unlike earlier models, our cohort includes patients who received more contemporary treatments, such as immunotherapy.

## 5. Conclusions

In conclusion, patients with poor performance status, extraspinal metastases, and older age at surgery are at high risk for mortality within 180 days following spinal surgery for metastatic disease. Patients with a lower weight are also at risk for poor outcomes. These results warrant a multidisciplinary discussion regarding the benefit of surgery in patients with risk factors identified in this study.

## Figures and Tables

**Table 1:** Patient characteristics.

Characteristic	Total Patients
**Gender (Male)**	69/128 (54%)
**Ethnicity**	
White	114/128 (89%)
African American	14/128 (11%)
**Age at Diagnosis ^a^ (years)**	
<65	97/128 (76%)
65 or >	27/128 (21%
**Smoking Status ^b^**	
Never	61/128 (48%)
Former	36/128 (28%)
Current	29/128 (23%)
**BMI (kg/m^2^)**	
<20	17/128 (13%)
20–30	72/128 (56%)
30.01–40	28/128 (22%)
>40	11/28 (9%)
**Tumor Type**	
Bladder	4/128 (3%)
Breast	15/128 (12%)
Gastrointestinal	7/128 (5%)
Gynecologic	1/128 (1%)
Lung	31/128 (24%)
Lymphoma	1/128 (1%)
Melanoma	4/128 (3%)
Multiple Myeloma	8/128 (6%)
Other	7/128 (5%)
Prostate	7/128 (5%)
Renal Cell Carcinoma	21/128 (16%)
Sarcoma	15/128 (12%)
Thyroid	7/128 (5%)
**ECOG**	
0	26/128 (20%)
1	67/128 (52%)
2	25/128 (19%)
3	10/128 (8%)
**Extraspinal Metastases ^c^**	
Yes	66/128 (52%)
No	60/128 (47%)
**Age at Surgery (years)**	
<65	94/128 (73%)
65 or >	34/128 (27%)

^a^: 4 patients with unknown age at diagnosis, ^b^: 2 patients with unknown smoking history, ^c^: 2 patients with unknown extraspinal metastases.

**Table 2:** Univariate and multivariate logistic regression assessing risk factors for death at 180 days.

	Univariate		Multivariate	
Risk Factors	Odds Ratio (95% CI)	*p*-Value	Odds Ratio (95% CI)	*p*-Value
Age at Diagnosis		0.3882		
<65	-ref-			
≥65	1.52 (0.59–3.97)			
Age at Surgery		0.0200		0.0316
<65	-ref-		-ref-	
≥65	2.8 (1.18–6.68)		2.78 (1.09–7.07)	
Tumor Type ^a^		0.2889		
Hematologic	-ref-			
Breast	0.61 (0.01–39.94)			
Lung	8.02 (0.36–177.23)			
Other Solid	10.47 (0.48–226.1)			
Renal	4.89 (0.2–117.46)			
Sarcoma	7.44 (0.3–182.79)			
BMI		0.1599		
≤30	-ref-			
>30	0.49 (0.18–1.32)			
# Vertebrae Involved		0.5446		
1 to 4	-ref-			
≥5	1.38 (0.48–3.95)			
Extraspinal Mets		0.0299		0.0110
No	-ref-		-ref-	
Yes	2.64 (1.1–6.36)		3.44 (1.33–8.93)	
ECOG		0.0276		0.0397
0/1	-ref-		-ref-	
2/3	2.64 (1.11–6.27)		2.66 (1.05–6.77)	
Pre-Op Therapy		0.4166		
No	-ref-			
Yes	1.42 (0.61–3.28)			

# = number. ^a^ The 13 tumor types were consolidated into 6 groups: Breast, Lung, Renal, Sarcoma, Hematologic (Multiple Myeloma and Lymphoma), and Other Solid (Bladder, Gastrointestinal, Gynecologic, Prostate, Thyroid, Melanoma), and Other (head and neck [2] squamous cell carcinoma of unknown primary [2], neuroblastoma, neuroendocrine tumor of unknown primary, thymoma).

**Table 3:** Univariate and multivariate cox proportional hazard regression assessing risk factors for overall survival.

	Univariate		Multivariate	
Risk Factors	Hazard Ratio (95% CI)	*p*-Value	Hazard Ratio (95% CI)	*p*-Value
Age at Diagnosis		0.2277		0.0562
<65	-ref-		-ref-	
≥65	1.33 (0.84–2.12)		0.48 (0.23–1.02)	
Age at Surgery		0.0062		0.0016
<65	-ref-		-ref-	
≥65	1.82 (1.19–2.8)		3.3 (1.57–6.91)	
Tumor Type ^a^		0.0370		
Hematologic	-ref-			
Breast	9.63 (1.22–75.84)			
Lung	18.55 (2.51–136.97)			
Other Solid	17.28 (2.35–127.16)			
Renal	12.98 (1.72–97.75)			
Sarcoma	12.39 (1.59–96.56)			
BMI		0.0002		0.0008
≤30	-ref-		-ref-	
>30	0.4 (0.24–0.65)		0.4 (0.23–0.68)	
# Vertebrae Involved		0.2553		
1 to 4	-ref-			
≥5	1.34 (0.81–2.21)			
Extraspinal Mets		0.0014		0.0001
No	-ref-		-ref-	
Yes	1.95 (1.29–2.93)		2.41 (1.54–3.77)	
ECOG		0.0001		0.0006
0/1	-ref-		-ref-	
2/3	2.35 (1.52–3.63)		2.36 (1.45–3.85)	
Pre-Op Therapy		0.4178		
No	-ref-			
Yes	1.18 (0.79–1.77)			

# = number. ^a^ The 13 tumor types were consolidated into 6 groups: Breast, Lung, Renal, Sarcoma, Hematologic (Multiple Myeloma and Lymphoma), and Other Solid (Bladder, Gastrointestinal, Gynecologic, Prostate, Thyroid, Melanoma), and Other (head and neck [2] squamous cell carcinoma of unknown primary [2], neuroblastoma, neuroendocrine tumor of unknown primary, thymoma).

## Data Availability

The data underlying this article will be shared upon reasonable request to the corresponding author.

## Supplementary Materials

The following supporting information can be downloaded at: https://www.mdpi.com/article/10.3390/cancers16020438/s1. Table S1: Preoperative Characteristics. Table S2: Surgical and Peri-Surgical Characteristics. Table S3: Characteristics of Receipt of Post-Operative Therapy. Table S4: Neurologic Outcomes Following Surgery. Table S5: Univariate and Multivariate Logistic Regressions Assessing Risk Factors Associated with Post-Op Therapy. Figure S1: Overall Survival in Patients Stratified by Tumor Type. Figure S2: Overall Survival in Patients Stratified by Age at Diagnosis. Figure S3: Overall Survival in Patients Stratified by Number of Vertebrae Involved. Figure S4: Overall Survival in Patients Stratified by Receipt of Pre-Operative Therapy.
