# Supplementary material for: Outcomes in Patients with Spinal Metastases Managed with Surgical Intervention
Source: Cancers (Basel). 2024 Jan 19;16(2):438. doi: 10.3390/cancers16020438 (PMC10813971; doi:10.3390/cancers16020438)
Supplement: Supplementary file 1 [file cancers-16-00438-s001.zip › Supplementary Figures S1-S4.pdf]

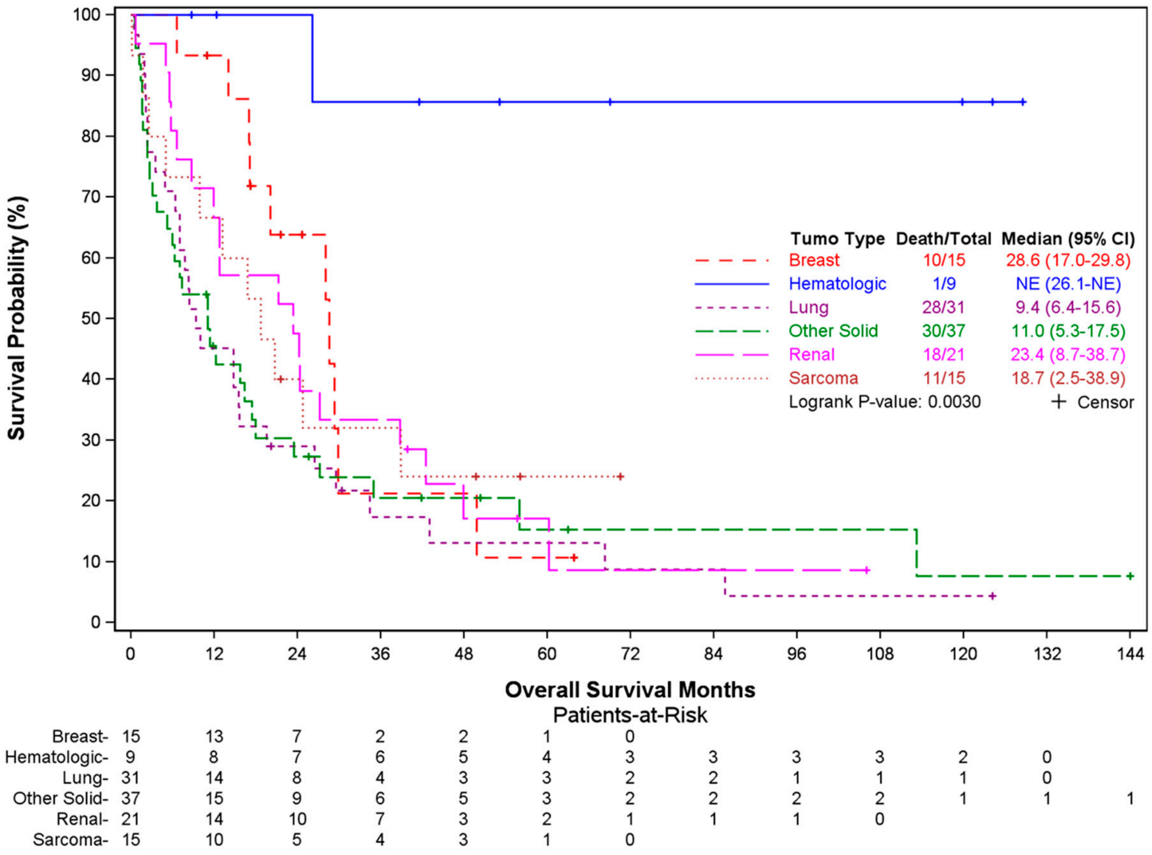

Figure S1: Overall Survival in Patients Stratified by Tumor Type.

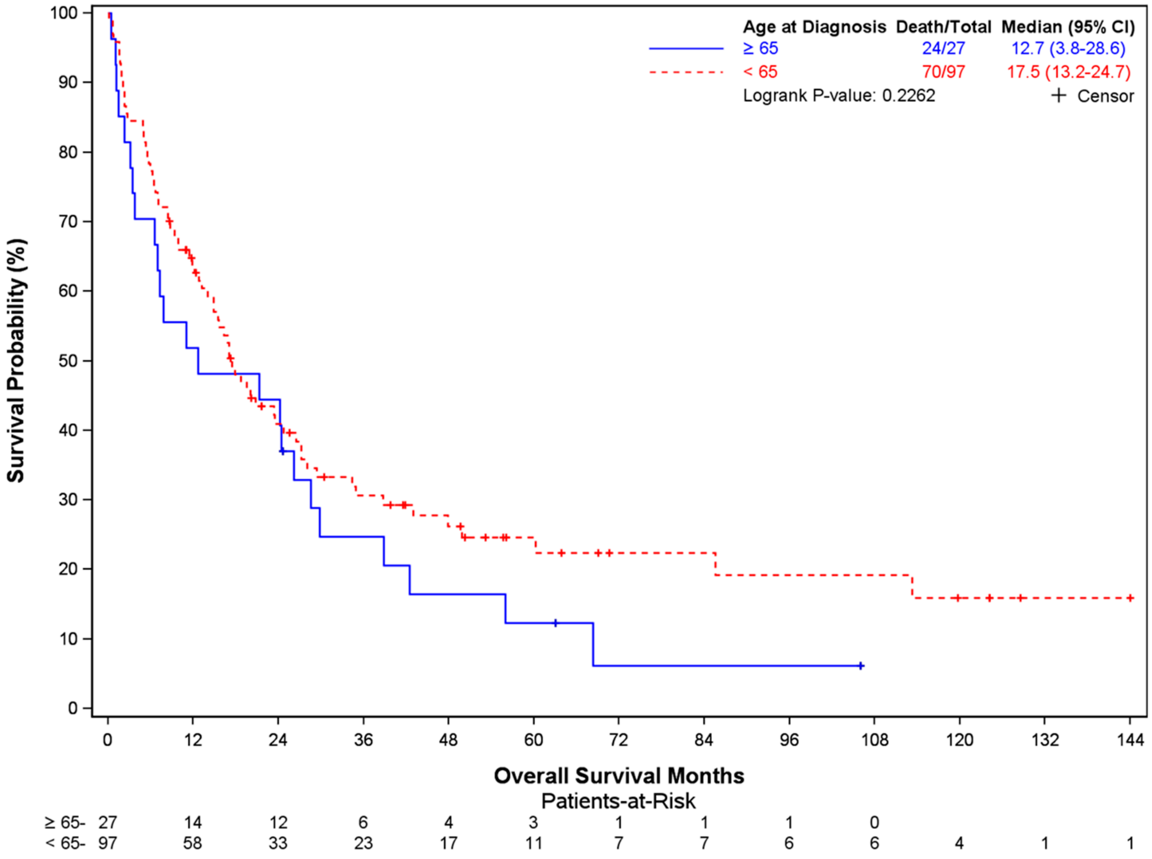

Figure S2: Overall Survival in Patients Stratified by Age at Diagnosis.

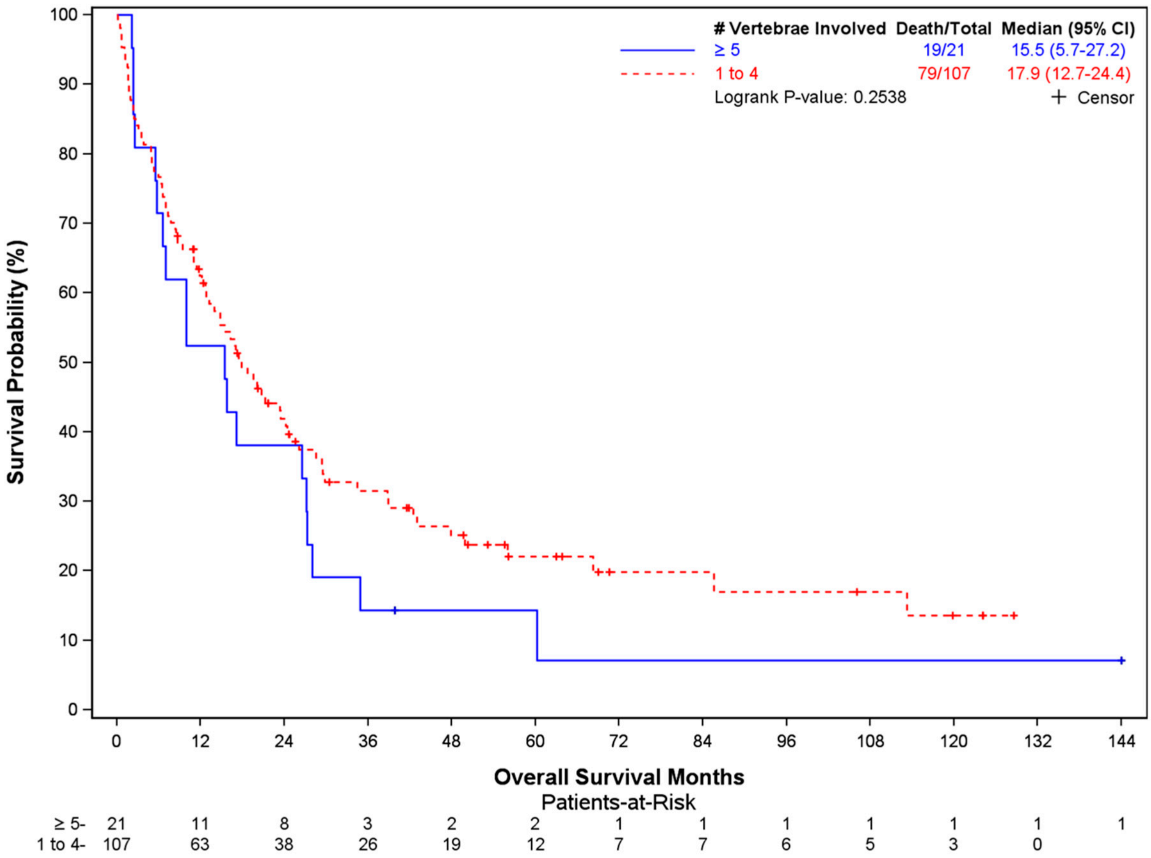

Figure S3: Overall Survival in Patients Stratified by Number of Vertebrae Involved.

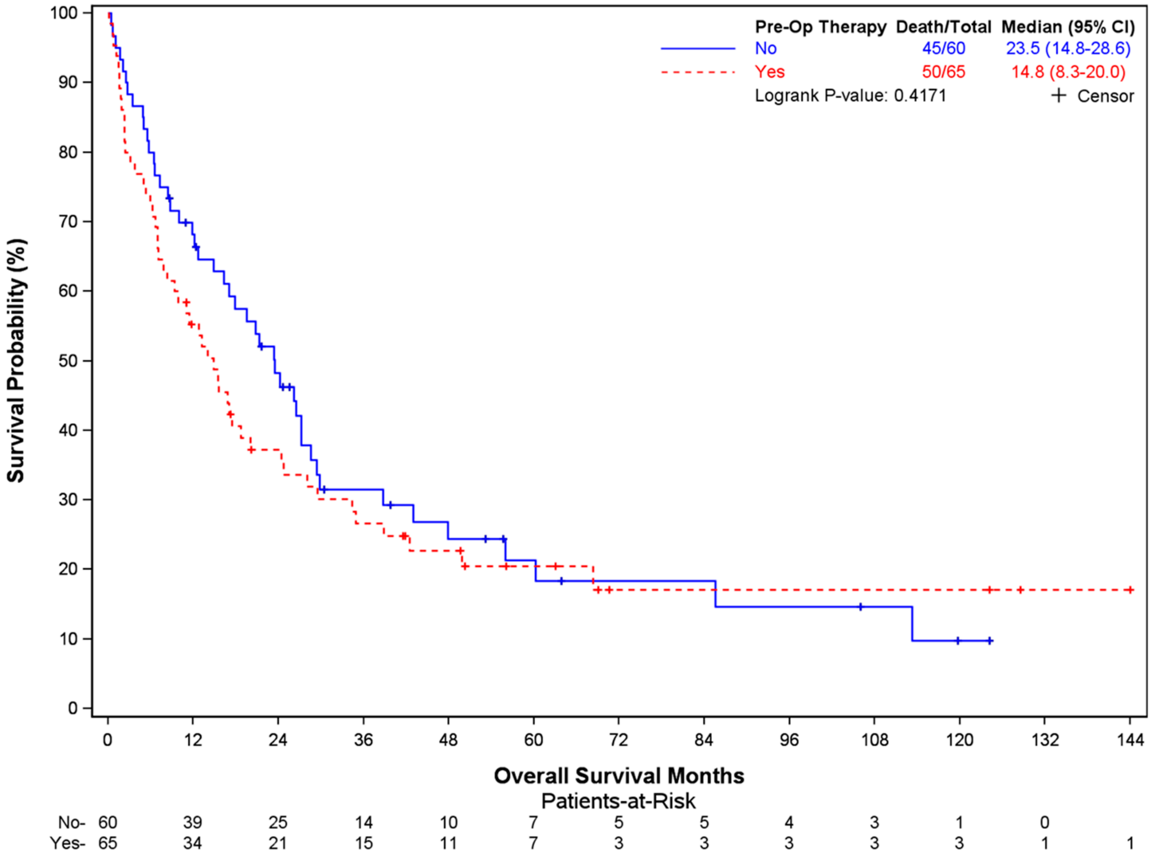

Figure S4: Overall Survival in Patients Stratified by Receipt of Pre-Operative Therapy.
