# Supplementary material for: Outcomes in Patients with Spinal Metastases Managed with Surgical Intervention
Source: Cancers (Basel). 2024 Jan 19;16(2):438. doi: 10.3390/cancers16020438 (PMC10813971; doi:10.3390/cancers16020438)
Supplement: Supplementary file 1 [file cancers-16-00438-s001.zip › Supplementary Tables S1-S5.pdf]

Supplementary Table S1: Preoperative Characteristics

Characteristic	Total Patients
**Number of Vertebrae with Metastases**	
1	37/128 (29%)
2 to 4	70/128 (55%)
5 to 10	15/128 (12%)
> 10	6/128 (5%)
**Location of Metastases**	
Cervical	32/128 (25%)
Thoracic	91/128 (71%)
Lumbar	32/128 (25%)
Sacral	5/128 (4%)
**Radicular Symptoms**	
Yes	68/128 (53%)
No	53/128 (41%)
Unknown	7/128 (5%)
**Serum Albumin**	
< 3.5	38/128 (30%)
3.5 or >	77/128 (60%)
**Received Radiation Therapy**	
Yes	59/128 (46%)
No	66/128 (52%)
Unknown	3/128 (2%)
**Received Immunotherapy**	
Yes	6/128 (5%)
No	118/128 (92%)
Unknown	4/128 (3%)
**Received Cytotoxic Therapy**	
Yes	56/128 (44%)
No	69/128 (54%)
Unknown	3/128 (2%)
**Received Other Therapy**[a]	
Yes	19/128 (15%)
No	104/128 (81%)
Unknown	5/128 (4%)
**Surgery on Primary Tumor**	
Yes	48/128 (38%)
No	70/128 (55%)
Unknown	10/128 (8%)
**Received Steroids Pre-operatively**	
Yes	30/128 (23%)
No	80/128 (63%)
Unknown	18/128 (14%)

a: includes miscellaneous therapies such as EGFR inhibitors, other tyrosine kinase inhibitors, oral hormonal therapy, etc

Supplementary Table S2: Surgical and Peri-Surgical Characteristics

Characteristic	Total Patients	Characteristic	Total Patients
**Surgical Approach**		**Postoperative Number of Transfusions**	
Anterior	7/128 (5%)	0	35/128 (27%)
Posterior	46/128 (36%)	1 to 2	41/128 (32%)
Combined Anterior Posterior	73/128 (57%)	3 to 5	28/128 (22%)
Unknown	2/128 (2%)	>6	8/128 (6%)
**Location of Vertebrae Decompressed**		Unknown	16/128 (13%)
Cervical	19/128 (15%)	**Length of Surgery (hours)**	
Cervical and thoracic	3/128 (2%)	≤ 3	27/128 (21%)
Thoracic	73/128 (57%)	3 to 5	38/128 (30%)
Thoracic and lumbar	13/128 (10%)	>5	11/128 (9%)
Lumbar	9/128 (7%)	Unknown	52/128 (22%)
Lumbar and sacral	3/128 (2%)	**Length of Hospital Stay (Days)**	
Unknown	8/128 (6%)	≤ 3	17/128 (13%)
**Number of Vertebrae Decompressed**		4 to 7	67/128 (52%)
1	32/128 (25%)	8 to 14	30/128 (23%)
2 to 3	68/128 (53%)	>14	6/128 (5%)
4 to 6	24/128 (19%)	Unknown	8/128 (6%)
Unknown	4/128 (3%)	**Additional Spinal Surgery**	
**Location of Vertebrae Instrumented**		Yes	23/128 (18%)
Cervical	15/128 (12%)	No	53/128 (41%)
Cervical and thoracic	24/128 (19%)	Unknown	52/128 (41%)
Thoracic	43/128 (34%)	**Additional Spinal Procedure**	
Thoracic and lumbar	29/128 (23%)	Cement Augmentation	6/128 (5%)
Lumbar	4/128 (3%)	Ablation	7/128 (5%)
Lumbar and sacral	7/128 (5%)	Unknown	17/128 (13%)
Sacrum	1/128 (1%)	**Postoperative Infection**	
Unknown	5/128 (4%)	Yes	17/128 (13%)
**Surgical Graft/Cage Use**		No	103/128 (80%)
None	22/128 (17%)	Unknown	8/128 (6%)
Cage	28/128 (22%)	**Other Postoperative Complication**	
Cement	26/128 (20%)	Yes	34/128 (27%)
Unknown	48/128 (38%)	Neurologic	8/34 (24%)
**Number of Vertebral Levels Instrumented**		DVT/PE	8/34 (24%)
1 to 3	19/128 (15%)	Wound	8/34 (24%)
4 to 6	68/128 (53%)	Cardiac/Respiratory	6/34 (18%)
>6	39/128 (30%)	No	79/128 (62%)
Unknown	2/128 (2%)	Unknown	15/128 (12%)
		**Death Within 30 days of Surgery**	4/128 (3%)

Supplementary Table S3: Characteristics of Receipt of Post-Operative Therapy

Characteristic	Total Patients
**Received Cytotoxic Therapy**	
Yes	53/128 (41%)
No	58/128 (45%)
Unknown	17/128 (13%)
**Received Radiation Therapy**	
Yes	78/128 (61%)
No	40/128 (31%)
Unknown	10/128 (8%)
**Received Immunotherapy**	
Yes	15/128 (12%)
No	96/128 (75%)
Unknown	17/128 (13%)
**Received Other Therapy**[a]	
Yes	42/128 (33%)
No	71/128 (55%)
Unknown	15/128 (12%)

a: includes therapies such as EGFR inhibitors, oral tyrosine kinase inhibitors, oral hormonal therapy, etc

Supplementary Table S4: Neurologic Outcomes Following Surgery

	Number of Patients		
**Neurologic Symptom/Deficit**[a]	**Pre-operative**	**Post-operative**[b]	**1 – 3 mo Post-operative**[c]
**Pain**	67/75 (89%)		
Stable/Improved		64/67 (96%)	53/67 (79%)
Worsened		3/67 (4%)	14/67 (21%)
**Motor**	21/75 (28%)		
Stable/improved		19/21 (90%)	15/21 (71%)
Worsened		2/21 (10%)	6/21 (29%)
**Sensory**	15/75 (20%)		
Stable/Improved		12/15 (80%)	12/15 (80%)
Worsened		3/15 (20%)	3/15 (20%)
**Asymptomatic**	4/75 (5.3%)		

[a] Detailed neurologic status was available for 75/128 patients pre-operatively. Immediate- and 1-3 mo post-operative status is reported as relative to their pre-operative neurologic status.

[b] Unknown in 1/75 patients.

[c] Unknown in 5 patients, 2 patients deceased. Known in 68/75 patients.

Supplementary Table S5: Univariate and Multivariate Logistic Regressions Assessing Risk Factors Associated with Post-Op Therapy

	Univariate		Multivariate	
**Risk Factors**	**Odds Ratio (95% CI)**	***p*-value**	**Odds Ratio (95% CI)**	***p*-value**
**Age at Diagnosis**		0.7074		
< 65	-ref-			
≥ 65	1.2 (0.46-3.11)			
**Age at Surgery**		0.1223		
< 65	-ref-			
≥ 65	0.51 (0.21-1.2)			
**Tumor Type** [a]		0.0582		0.0491
Hematologic	-ref-		-ref-	
Breast	1.86 (0.1-34.43)		1.77 (0.1-32.84)	
Lung	0.14 (0.02-1.35)		0.12 (0.01-1.12)	
Other Solid	0.17 (0.02-1.53)		0.15 (0.02-1.35)	
Renal	0.5 (0.05-5.36)		0.4 (0.04-4.34)	
Sarcoma	0.14 (0.01-1.49)		0.12 (0.01-1.31)	
**BMI**		0.4729		
≤ 30	-ref-			
>30	1.36 (0.58-3.18)			
# **Vertebrae Involved**		0.1841		0.1041
1 to 4	-ref-		-ref-	
≥ 5	2.23 (0.68-7.3)		2.78 (0.81-9.55)	
**Extraspinal Mets**		0.4881		
No	-ref-			
Yes	0.76 (0.35-1.65)			
**ECOG**		0.0593		
0/1	-ref-			
2/3	0.45 (0.19-1.03)			
**Pre-Op Therapy**		0.5821		
No	-ref-			
Yes	0.8 (0.37-1.74)			

[a] The 13 tumor types were consolidated into 6 groups: Breast, Lung, Renal, Sarcoma, Hematologic (Multiple Myeloma and Lymphoma), and Other solid (Bladder, Gastrointestinal, Gynecologic, Prostate, Thyroid, Melanoma, and Other (head and neck [2] squamous cell carcinoma of unknown primary [2], neuroblastoma, neuroendocrine tumor of unknown primary, thymoma)
